# Susceptibility of hepatoma-derived cells to histone deacetylase inhibitors is associated with *ID2* expression

**DOI:** 10.3892/ijo.2013.1811

**Published:** 2013-02-06

**Authors:** RYOUICHI TSUNEDOMI, NORIO IIZUKA, SAWAKO HARADA, MASAAKI OKA

**Affiliations:** 1 Departments of Digestive Surgery and Surgical Oncology, Yamaguchi University Graduate School of Medicine, Ube, Yamaguchi 755-8505, Japan; 2 Complementary Medicine, Yamaguchi University Graduate School of Medicine, Ube, Yamaguchi 755-8505, Japan

**Keywords:** inhibitor of DNA binding 2, hepatocellular carcinoma, histone deacethylase inhibitor, apoptosis

## Abstract

Downregulation of *inhibitor of DNA binding 2* (*ID2*) is associated with poor prognosis in cases of hepatocellular carcinoma (HCC). Therefore, to search for effective antitumor drugs for the treatment of HCC exhibiting poor prognostic indicators, we used two HCC-derived cell lines (HuH-7 and HLE) to alter *ID2* levels. Specifically, *ID2* expression was knocked down in HuH-7 cells via transfection with *ID2*-specific small interfering RNAs and separately *ID2* was overexpressed in HLE cells via an *ID2* expression plasmid vector. To assess the effect of antitumor drugs, MTS assay was performed. Annexin V staining was used to evaluate apoptosis and real-time RT-PCR was used to measure mRNA levels. *ID2* knockdown cells were more susceptible to histone deacethylase (HDAC) inhibitors including sodium butyrate (NaB), sodium 4-phenyl-butyrate, tricostatin A, suberoylanilide hydroxamic acid, MS-275, apicidin and HC-toxin. Conversely, cells that overexpressed *ID2* were less susceptible than control cells to HDAC inhibitors. NaB-induced apoptosis was inversely correlated with *ID2* expression. Expression of the anti-apoptotic mRNA *BCL2* was induced by NaB in control cells, but this induction of *BCL2* was inhibited by *ID2* knockdown and strengthened by *ID2* overexpression. Expression of another anti-apoptotic mRNA, *BCL2L1*, was decreased by NaB administration and then partially recovered. However, in *ID2* knockdown cells, *BCL2L1* levels did not recover from NaB-induced suppression. *ID2* affected the susceptibility of two HCC-derived cell lines to an HDAC inhibitor by regulating the expression of anti-apoptotic genes. Therefore, HDAC inhibitors may be effective for the treatment of HCC for which the prognosis is poor based on *ID2* downregulation and *ID2* could serve as a marker that is predictive of the clinical response to HDAC inhibitors.

## Introduction

Hepatocellular carcinoma (HCC) is one of the most lethal malignancies worldwide (1,2). HCC is caused mainly by chronic liver inflammation due to hepatitis B virus, hepatitis C virus (HCV) or alcohol abuse (1). Despite curative resection and recent advances in treatments, the clinical course of HCC is variable and many patients suffer recurrence after surgery. Poor prognoses in cases of HCC can be explained largely by the high rate of intrahepatic recurrence (IHR), which results from metastatic spread of cancer cells (3).

Previously, we identified a gene, *inhibitor of DNA binding 2* (*ID2*), that is significantly downregulated in HCCs, especially in advanced HCCs, relative to surrounding liver tissues (4,5). Moreover, we found that *ID2* is a portal vein invasion-related gene in HCV-related HCC (6) and that *ID2* negatively regulates the invasive potential of cancer cells (7). Therefore, HCC patients with low *ID2* expression have poor prognoses (7). *ID2* belongs to a protein family that comprises ID1 to ID4; these proteins have a helix-loop-helix structure and form heterodimers with basic helix-loop-helix transcription factors to act as dominant-negative inhibitors of transcription (8–10). IDs are involved in proliferation processes, differentiation, development, senescence and angiogenesis (11–15), and are linked to various malignant tumors (16–31).

In this study, we searched for antitumor drugs that are effective against cells with low *ID2* expression because such antitumor drugs might be useful in the treatment of patients who have HCC and a poor prognosis. We found that alteration of *ID2* expression affected the susceptibility of cells to histone deacetylase (HDAC) inhibitors and that HDAC inhibitors were the only antitumor drugs tested for which alteration of *ID2* expression had an effect. HDAC inhibitors have emerged as a new class of antitumor agents (32–34). HDAC inhibitors can cause multiple epigenetic changes in aberrant cells. Treatment with HDAC inhibitors most frequently induces apoptosis (35–37). Although their precise mode of action remains uncertain, HDAC inhibitors can modulate the cell cycle, apoptosis, angiogenesis, invasion and metastases (32,33,38–40). Here, we aimed to investigate how and whether *ID2* affected the anti-tumor activity of sodium butyrate (NaB), an HDAC inhibitor.

## Materials and methods

### Hepatoma cell lines

Human hepatoma-derived cell lines, HLE and HuH-7, were purchased from the Health Science Research Resources Bank (Osaka, Japan). Cells were cultured in DMEM (Nissui Pharmaceutical, Tokyo, Japan) containing 10% heat-inactivated fetal bovine serum (Life Technologies, Tokyo, Japan) and supplemented with penicillin (100 U/ml), streptomycin (100 *μ*g/ml) and sodium bicarbonate (1.5 g/l) at 37°C in 5% CO_2_ in air. As in our previous report (7), *ID2*-knockdown and *ID2*-overexpression were accomplished by transfection of HuH-7 and HLE cells with *ID2*-specific small interfering RNAs (siRNAs) or an *ID2*-expression plasmid vector, respectively. HuH-7 and HLE cells transfected with control siRNA or empty pcDNA3.1(-) plasmid DNA were used as the respective control.

### Administration of histone deacetylase inhibitors

NaB (Sigma-Aldrich, Tokyo, Japan), sodium 4-phenyl-butyrate (NaPB; Funakoshi, Tokyo, Japan), tricostatin A (TSA; Sigma-Aldrich), suberoylanilide hydroxamic acid (SAHA; Cosmo Bio, Tokyo, Japan), MS-275 (Sigma-Aldrich), apicidin (Sigma-Aldrich) and HC-toxin (Sigma-Aldrich) were each used as an HDAC inhibitor in this study. HDAC inhibitors were added to cultures 24 h after cells had been seeded; cultures were then further incubated with the inhibitor for defined periods at 37°C in 5% CO_2_ in air.

### MTS assay

The CellTiter 96 AQueous One Solution Cell Proliferation Assay (Promega, Tokyo, Japan) which includes 3-(4,5-dimethylthiazol-2-yl)-5-(3-carboxymethoxyphenyl)-2-(4-sulfophenyl)-2H-tetrazolium, inner salt (MTS) was used according to the manufacturer’s instructions to evaluate cell survival. Cells (3×10^3^) were seeded into the wells of 96-well plates and cultivated. At the appropriate time, MTS was added to the cells, which were then incubated for 2 additional hours at 37°C. The optical density of the culture medium at 492 and 650 nm were then measured by using an EnVision plate reader (PerkinElmer, Waltham, MA). Triplicate wells were analyzed in each assay.

### Annexin V staining

The Annexin V-FLUOS Staining Kit (Roche Diagnostics, Tokyo, Japan), which includes Annexin V/propidium iodide (PI), was used according to the manufacturer’s instructions to detect apoptosis. The cultures were observed under a fluorescent microscope (IX71; Olympus, Tokyo, Japan). Simultaneously, Hoechst33342-positive cells were counted as total number of cells in cultures; Hoechst33342 was purchased from Sigma-Aldrich.

### Semiquantitative real-time RT-PCR

Semiquantitative real-time RT-PCR (semi-qRT-PCR) was performed as described previously (7,41) with minor modifications. Real-time PCR amplification was performed using the LightCycler 480 Probe Master and Universal ProbeLibrary Probes in a LightCycler System Version 3 (all from Roche Diagnostics). Primers and probes that were used are listed in Table I. Amplification was performed according to a two-step cycle procedure consisting of 45 cycles of denaturation at 95°C for 10 sec and annealing/elongation at 60°C for 30 sec. We used the Δ/Δ threshold cycle method to semiquantitatively measure mRNA levels; *glyceraldehyde-3-phosphate dehydrogenase* (*GAPDH*) and *β-actin* (*ACTB*) were both used as reference genes. All values of mRNA levels are expressed relative to control.

### Statistical analysis

Data are presented as mean ± standard deviation. Dunnett’s test for multiple comparisons was used to evaluate the differences between three groups. Calculations were performed using SPSS Statics 17.0 software (IBM, Tokyo, Japan). P<0.05 was considered statistically significant.

## Results

### Susceptibility to histone deacetylase inhibitors in HCC-derived cell lines in which ID2 was knocked down or overexpressed

**Figure 1:**
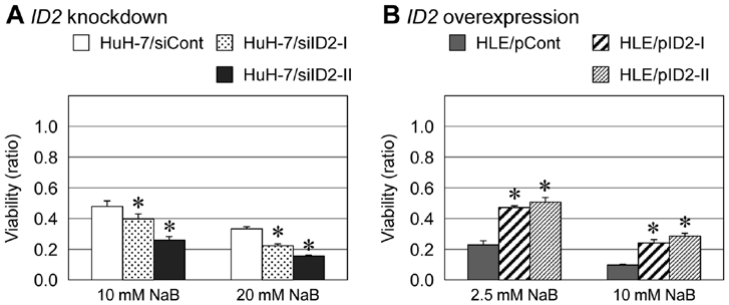
*ID2* levels and antitumor activity of NaB. Cells were subjected to an MTS assay 72 h after 20 mM NaB administration; NaB is one of several HDAC inhibitors that had an effect on survival of HCC-derived cells. Cell viability was lower in HCC-derived cells transfected with *ID2* knockdown siRNAs than those transfected with control siRNA. Cell viability was higher in HCC-derived cells that overexpressed *ID2* than in those transfected with an empty vector. ^*^P<0.05 compared with HuH-7/siCont or HLE/pCont.

**Figure 2:**
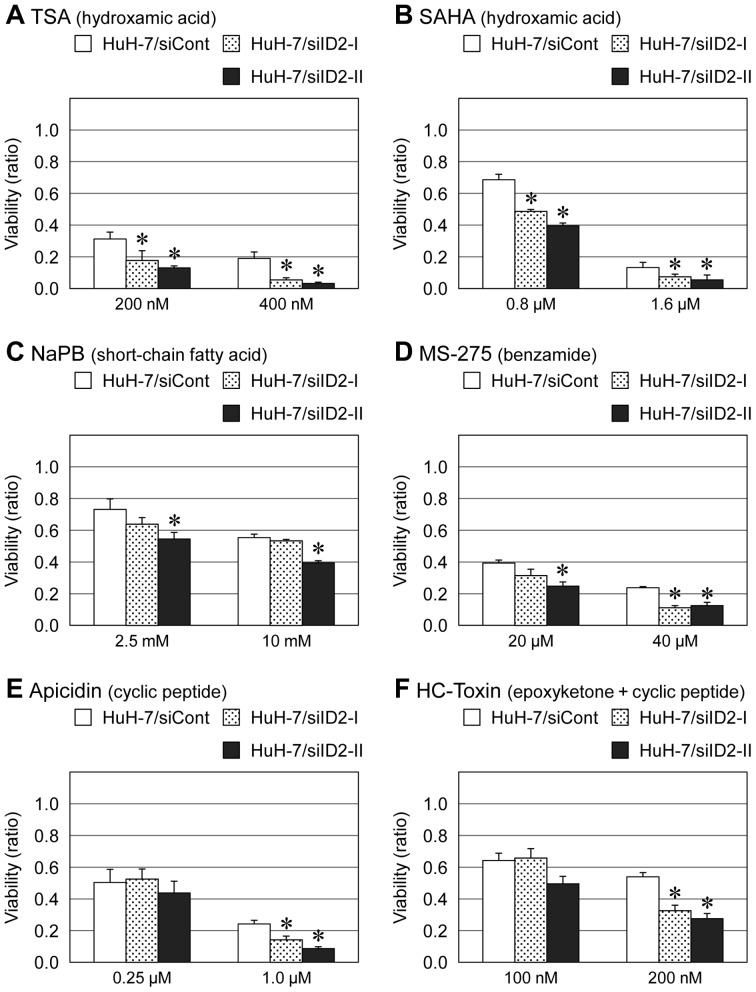
The antitumor activity of HDAC inhibitors in *ID2* knockdown cells. Cells were subjected to an MTS assay to evaluate the effect of *ID2* on the antitumor activity of HDAC inhibitors other than NaB. Each HDAC inhibitor had an effect similar to that of NaB (Fig. 1) on the *ID2* knockdown cells. ^*^P<0.05 compared with HuH-7/siCont.

**Figure 3:**
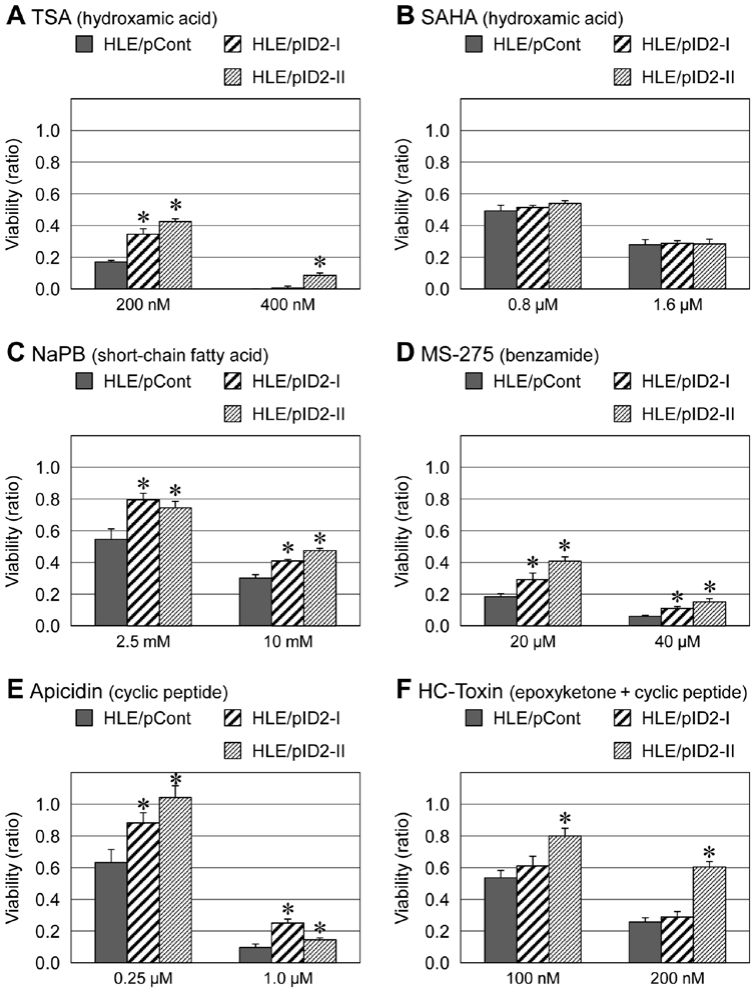
The antitumor activity of HDAC inhibitors in cells that overexpressed *ID2*. Cells were subjected to an MTS assay to evaluate the effect of *ID2* on the antitumor activity of HDAC inhibitors other than NaB. In cells that overexpressed *ID2*, each HDAC inhibitor, except SAHA, had an effect similar to that of NaB (Fig. 1). ^*^P<0.05 compared with HLE/pCont.

**Figure 4:**
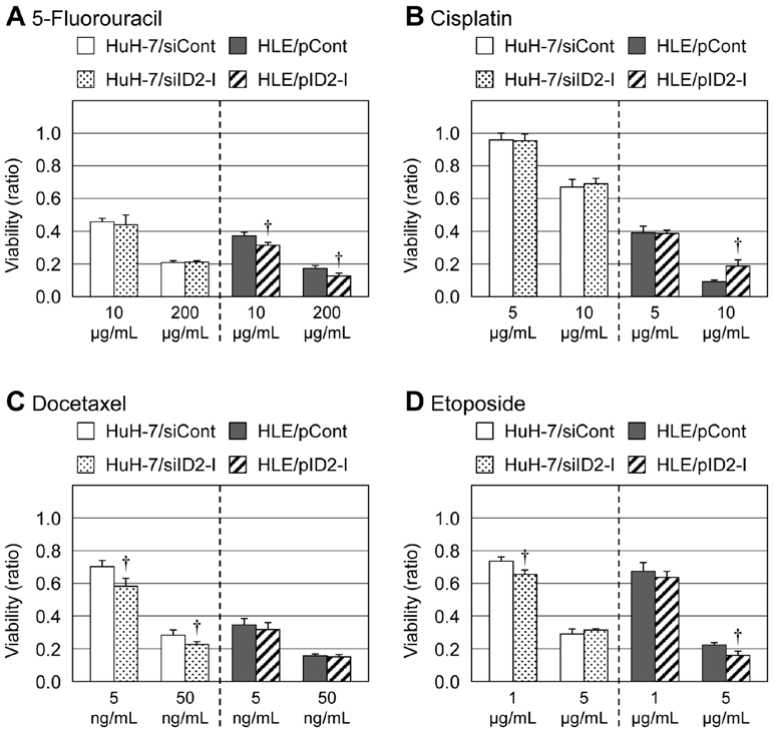
*ID2* levels and antitumor activity. Cells were subjected to MTS assay 72 h after administration of the indicated antitumor drugs,. ^†^P<0.05 compared with HuH-7/siCont or HLE/pCont.

We used the MTS assay and previously established HCC-derived cell lines in which *ID2* expression was suppressed or enhanced (7) to examine the susceptibility of HCC cells to antitumor drugs. Among the tested antitumor drugs, the antitumor activity of an HDAC inhibitor, NaB, was increased in *ID2* knockdown cells and decreased in *ID2*-overexpressing cells (Fig. 1). Similar results were obtained with other HDAC inhibitors including TSA, SAHA, PBA, MS-275, apicidin and HC-toxin (Figs. 2 and 3). However, for other types of antitumor agents than HDAC inhibitors (e.g., 5-fluorouracil, cisplatin, docetaxel and etoposide), such results were not observed (Fig. 4).

### Influence of ID2 on NaB-induced apoptosis

**Figure 5:**
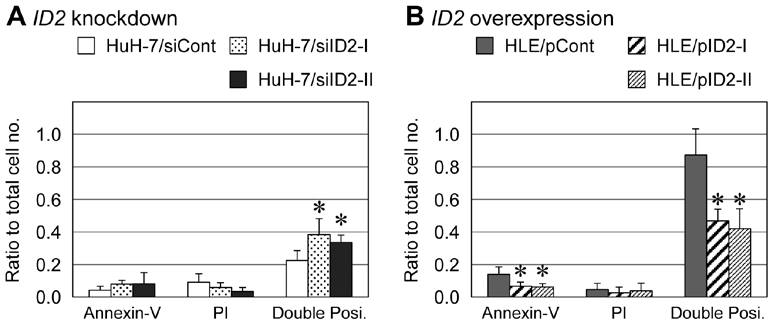
*ID2* levels and apoptosis caused by NaB. Cells were stained with Annexin V/Propidium iodide (PI)/Hoechst 33342 after 20 mM NaB had been administered for 72 h; cells were then assessed by fluorescence microscope. Cells positive for both Annexin V and PI staining were considered to be in the late stage of apoptosis. Cultures containing *ID2* knockdown cells had higher percentages of apoptotic cells than did cultures with control siRNA transfected cells. Cultures with *ID2* overexpressing cells had lower percentages of apoptotic cells than did cultures containing cells transfected with empty vector. ^*^P<0.05 compared with HuH-7/siCont or HLE/pCont.

In HLE derivatives treated with 20 mM NaB for 72 h, the number of cells positive for both Annexin V and PI (late apoptosis) was significantly lower among *ID2*-overexpressing cells than empty-vector control cells (approximately 44 vs. 87%, respectively) (Fig. 5B). For HuH-7 derivatives treated with 20 mM NaB for 72 h, the percentage of cells positive for both Annexin V and PI (late apoptosis) was 34% among *ID2*-knockdown cells and 25% among siRNA-transfected control cells (Fig. 5A). In both HLE and HuH-7 derivatives, Annexin V single-positive cells (early apoptosis) showed same tendency with the Annexin V and PI double-positive cells, although Annexin V single-positive cells were less than 10% of each cell type.

**Figure 6:**
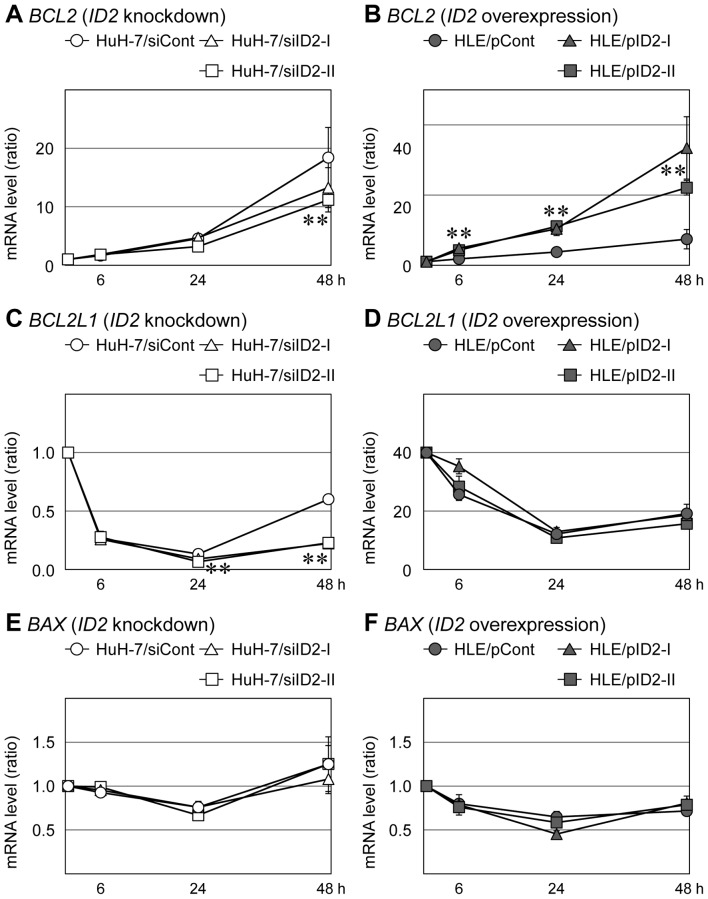
Changes in expression of anti-apoptotic genes following NaB administration. The mRNA levels of BCL2, BCL2L1 and BAX were measured after 20 mM NaB administration for 0, 6, 24 or 48 h. (A and B) BCL2 mRNA level was induced by NaB administration when compared to the induction in control cells, this NaB-dependent induction was suppressed in *ID2* knockdown cells and enhanced in cells that overexpressed *ID2*. (C and D) The BCL2L1 mRNA level decreased immediately after NaB administration and was then partially restored. In *ID2* knockdown cells, the restoration of BCL2L1 expression was largely suppressed relative to that in control cells. ^**^Both ID2-targeted cells showed P<0.05 compared with control cells.

We examined expression of apoptosis-related genes in HLE and HuH-7 cells that had been treated with NaB. Following addition of 20 mM NaB, about half of the HLE cells had died within 24 h and about half of the HuH-7 cells had died within 48 h. Treatment with NaB induce expression of *BCL2* mRNA (an anti-apoptotic mRNA) in HuH-7 cells transfected with control siRNA and in HLE cells transfected with empty vector; however, this NaB-dependent induction was suppressed in HuH-7 cells transfected with *ID2*-specific siRNAs and enhanced in HLE cells that overexpressed *ID2* (Fig. 6A and B). Levels of another anti-apoptotic mRNA, *BCL2L1* (*BCL-XL*), decreased immediately after addition of NaB in control HuH-7 cells and in *ID2*-knockdown HuH-7 cells; 48 h after NaB addition, *BCL2L1* levels had partially recovered in control cells, but they had not recovered in *ID2*-knockdown HuH-7 cells (Fig. 6C). HLE cells that overexpressed *ID2* and control HLE cells did not differ significantly in *BCL2L1* mRNA levels (Fig. 6D). The mRNA level of *BAX,* a pro-apoptotic gene, was not influenced by *ID2* expression (Fig. 6E and F).

## Discussion

In this study, *ID2* negatively regulated the susceptibility of HCC-derived cells to HDAC inhibitors (Figs. 1–3). Several types of antitumor drugs were tested for their effects on HCC-derived cells with altered *ID2* levels; cells became more susceptible HDAC inhibitors when *ID2* was downregulated, but no other type of antitumor drug had this effect (Fig. 4). Previous reports showed that *ID2* expression was linked to poor prognosis of HCC (5–7). *ID2* expression is low in HCC samples that also exhibit other poor prognosis indicators such as poor differentiation or portal vein invasion. Therefore, we reasoned that HDAC inhibitors may be useful for treating HCC characterized by indicators of poor prognosis.

HDAC inhibitors have emerged as a new class of antitumor drugs that are intended to modulate epigenetic regulation and several clinical trials have been conducted (42,43). Although HDAC inhibitors act on HDACs specifically, genome-wide acetylation of chromatin as a result of HDAC inhibition cause changes in the expression of many genes (45–47). Interestingly, addition of NaB to HCC-derived cell lines induced expression of anti-apoptotic *BCL2* and this induction of *BCL2* was positively regulated by *ID2* expression (Fig. 6). This finding indicates that *ID2* may exert an anti-apoptotic function via regulation of anti-apoptotic genes in the presence of this HDAC inhibitor (Figs. 5 and 6), although a pro-apoptotic gene, *BAX*, was not influenced by *ID2* expression (Fig. 6). *Cyclin-dependent kinase inhibitor 1A* (*CDKN1A; p21*, *Cip1*) is one of the genes activated by HDAC inhibitors and activated *CDKN1A* inhibits the transition from G1 to S phase (48). The activation of *CDKN1A* induced by HDAC inhibitors results in growth arrest and apoptosis in several malignant cell types (47,49,50). We also observed NaB-mediated induction of *CDKN1A* and this induction was significantly suppressed by *ID2* overexpression (data not shown). In *ID2* knockdown cells, however, that NaB-mediated *CDKN1A* induction was not affected. The expression of *ID2* itself was gradualy induced following the addition of NaB (data not shown). This increase in *ID2* expression might be an endogenous defensive effect in response to HDAC inhibitors. Because ID2 acts as a dominant-negative inhibitor of basic helix-loop-helix transcription factors by forming heterodimers (8–10), some of counter partners forming heterodimers with ID2 may responsible for HDAC susceptibility.

We suggest that *ID2* could serve as a predictive marker of the response of HCC to HDAC inhibitors. *ID2* influences the susceptibility of HCC cells to the HDAC inhibitor by regulating the expression of anti-apoptotic genes. Further investigation of the mechanism by which *ID2* affects susceptibility to HDAC inhibitors, and of the influence of *ID2* on DNA methylation are needed because histone acetylation and DNA methylation are each correlated with epigenetic regulation. Biomarkers for clinical response are strongly needed for improvement of patients’ quality of life and also medical economics. *ID2* may be useful as a biomarker of the likely response of HCC to HDAC inhibitors; moreover, further research on *ID2* expression in HCC may contribute to the identification of new molecular targets that can be altered to enhance the effects of HDAC inhibitors. Such advances may lead to the improvement of antitumor therapy that is based on HDAC inhibitors.

## Figures and Tables

**Table I:** The primers and hydrolysis probes used in this study.

Primers and probes	Sequence (5′→3′)
*ID2*	
5′-primer	ATATCAGCATCCTGTCCTTGC
3′-primer	AAAGAAATCATGAACACCGCTTA
Hydrolysis probe	UPL Probe #5^a^
*BCL2*	
5′-primer	TTGACAGAGGATCATGCTGTACTT
3′-primer	ATCTTTATTTCATGAGGCACGTT
Hydrolysis probe	UPL Probe #6^a^
*BCL2L1 (BCL-XL)*	
5′-primer	GCTGAGTTACCGGCATCC
3′-primer	AGATTCTGAAGGGAGAGAAAGAGA
Hydrolysis probe	UPL Probe #83^a^
*BAX*	
5′-primer	ATGTTTTCTGACGGCAACTTC
3′-primer	ATCAGTTCCGGCACCTTG
Hydrolysis probe	UPL Probe #57^a^
*CDKN1A (P21)*	
5′-primer	TCACTGTCTTGTACCCTTGTGC
3′-primer	GGCGTTTGGAGTGGTAGAAA
Hydrolysis probe	UPL Probe #32^a^
*GAPDH*	
5′-primer	AGCCACATCGCTCAGACAC
3′-primer	GCCCAATACGACCAAATCC
Hydrolysis probe	UPL Probe #60^a^
*ACTB*	
5′-primer	CCAACCGCGAGAAGATGA
3′-primer	CCAGAGGCGTACAGGGATAG
Hydrolysis probe	UPL Probe #64^a^

a The number of Universal ProbeLibrary probes (Roche Applied Bioscience).
